# Vestibular implant: does it really work? A systematic review^☆^

**DOI:** 10.1016/j.bjorl.2019.07.011

**Published:** 2019-09-20

**Authors:** Yaná Jinkings de Azevedo, Alleluia Lima Losno Ledesma, Larissa Vilela Pereira, Carlos Augusto Oliveira, Fayez Bahmad Junior

**Affiliations:** a Universidade de Brasília (UnB), Faculdade de Ciências de Saúde, Programa de Pós-Graduação em Ciências da Saúde, Brasília, DF, Brazil; b Universidade de Brasília (UnB), Programa de Ciências da Saúde, Brasília, DF, Brazil; c Centro Universitário Planalto do Distrito Federal (UNIPLAN), Curso de Fonoaudiologia, Brasília, DF, Brazil; d Faculdade de Ciências de Saúde ‒ Asa Norte, Brasília, DF, Brazil; e Universidade de São Paulo (USP), Hospital das Clínicas, São Paulo, SP, Brazil; f Hospital Anchieta, Brasília, DF, Brazil; g Hospital Universitário de Brasília, Brasília, DF, Brazil; h Universidade de Brasília (UnB), Brasília, DF, Brazil

**Keywords:** Vestibular loss, Vestibular implant, Vestibular function, Perda vestibular, Implante vestibular, Função vestibular

## Abstract

**Introduction:**

People with vestibular loss present a deficit in the vestibular system, which is primarily responsible for promoting postural control, gaze stabilization, and spatial orientation while the head moves. There is no effective treatment for a bilateral loss of vestibular function. Recently, a vestibular implant was developed for people with bilateral loss of vestibular function to improve this function and, consequently, the quality of life of these patients.

**Objective:**

To identify in the scientific literature evidence that vestibular implants in people with vestibular deficit improves vestibular function.

**Methods:**

One hundred and forty six articles were found from five databases and 323 articles from the gray literature mentioning the relationship between vestibular implant and vestibular function in humans. The PICOS strategy (Population, Intervention, Comparison and Outcome) was used to define the eligibility criteria. The studies that met the inclusion criteria for this second step were included in a qualitative synthesis, and each type of study was analyzed according to the bias risk assessment of the Joanna Briggs Institute through the critical assessment checklist Joanna Briggs institute for quasi-experimental studies and the Joanna Briggs institute critical assessment checklist for case reports.

**Results:**

Of the 21 articles included in reading the full text, 10 studies were selected for the qualitative analysis in the present systematic review. All ten articles analyzed through the critical assessment checklist Joanna Briggs institute showed a low risk of bias. The total number of samples in the evaluated articles was 18 patients with vestibular implants.

**Conclusions:**

Taken together, these findings support the feasibility of vestibular implant for restoration of the vestibulo-ocular reflex in a broad frequency range and illustrate new challenges for the development of this technology.

## Resumo

**Introdução:**

Pessoas com perda vestibular apresentam um déficit no sistema vestibular, o qual é o principal responsável pelo controle postural, pela estabilização do olhar e orientação espacial enquanto a cabeça se movimenta. Não há tratamento efetivo para uma perda vestibular bilateral. Recentemente, foi desenvolvido um implante vestibular para pessoas com perda vestibular bilateral para melhorar essa função e, consequentemente, a qualidade de vida desses pacientes.

**Objetivo:**

Identificar na literatura científica evidências de que o implante vestibular melhora a função vestibular de pessoas com déficit vestibular.

**Método:**

Cento e quarenta e seis artigos foram encontrados em cinco bases de dados e 323 artigos da literatura cinzenta, mencionando a relação entre implante vestibular e função vestibular em humanos. A estratégia PICOS (População, Intervenção, Comparação e Desfechos) foi utilizada para definir os critérios de elegibilidade. Os estudos que preencheram os critérios de inclusão para esta segunda etapa foram incluídos em uma síntese qualitativa, e cada tipo de estudo foi analisado de acordo com a avaliação de risco de viés do Joanna Briggs Institute através da *critical appraisal checklist for quasi-experimental studies* e da *critical appraisal checklist for case reports*.

**Resultados:**

Dos 21 artigos incluídos cujos textos completos foram lidos, 10 foram selecionados para a análise qualitativa na presente revisão sistemática. Todos os dez artigos analisados ​​através da *critical appraisal checklist* mostraram um baixo risco de viés. O número total de amostras nos artigos avaliados foi de 18 pacientes com implantes vestibulares.

**Conclusões:**

Em conjunto, esses achados apoiam a viabilidade do implante vestibular para a restauração do reflexo vestíbulo-ocular em uma ampla faixa de frequências e ilustram novos desafios para o desenvolvimento desta tecnologia.

## Introduction

Every day we are confronted with a variety of dynamic situations in which precise information on head and body motion and on the spatial positions of the head and body is required to guarantee adequate function of the vestibular system and, consequently, safety and well being.^1^ The vestibular system is one of the main systems responsible for ensuring postural control, stabilization of the gaze and a spatial orientation, contributing to the maintenance of balance.^2^

This system consists of a set of sensory organs located in the inner ear, where it is composed of three semicircular canals and two otolithic organs (saccule and utricle), responsible for providing the cortex with simultaneous information about the position of the head in relation to the body and producing the Vestibulo-Ocular Reflex (VOR), which has the function of generating compensatory ocular movements in relation to the direction of the head to maintain a stable field of view in the retina.^1^

If the system is damaged by disease, aging or injury, the person will experience a loss of vestibular function. As a result, an inefficient or absent VOR and, consequently, a reduction of visual acuity will occur during movement.^3^, ^4^ These vestibular deficits can cause symptoms such as oscillopsia, imbalance, vertigo, spatial disorientation, and cognitive alterations, among others,^5^ compromising the patient's ability to perform basic daily activities such as walking.^6^

Despite scientific advances, the existing therapeutic options are limited and of low effectiveness in certain cases.^7^ Studies show that, despite intensive balance training, some patients do not show significant improvement, maintaining disabling symptoms with interference in daily and work activities.^1^, ^3^, ^6^, ^8^ In this context, some research groups suggest a Vestibular Implant (VI) to replace the function of the vestibular organs.^8^, ^9^, ^10^, ^11^

### Vestibular implants

Currently, the use of VI in humans has been studied by research groups in Europe (Geneva University Hospitals and Maastricht University Medical Center) and the United States (University of Washington) as a therapeutic alternative for people with definitive vestibular loss without improvement with clinical treatment.^12^, ^13^ Research on both animals^10^, ^11^, ^14^, ^15^ and humans^8^, ^10^, ^12^ has shown that electrical stimulation is a potentially effective means of activating the vestibular system.

The VI consists of a modification of the Cochlear Implant (CI), containing one to three “vestibular” electrodes taken out of the cochlear electrode array, each constructed with an arrangement of 2.5 mm fine electrodes with a diameter of 150 μm, implanted in the perilymphatic space adjacent to the membranous labyrinth of each semicircular canal.^13^ Thus, rather than detecting sound information as the CI, it capture the information on movement using head-fixed sensors and fed to a special processor where it is converted into an appropriate neural pattern.^2^ This neural patterns of processed motion information are then transmitted in the form of electrical currents delivered via the vestibular electrodes to the implanted stimulator and transfers the information to the Central Nervous System (CNS).^2^, ^8^ In this concept, the VI aims at transmitting “artificial” neural patterns to the CNS similar to those coded by the normally functioning vestibular system.^2^

To date, two surgical strategies have been described for VI: the intralabyrinthine surgical approach^1^, ^8^, ^12^, ^16^ and the extralabyrinthine approach.^15^, ^17^, ^18^ With the intralabyrinthine approach, each semicircular canal is opened, and the electrodes are inserted until they contact the ampullary ciliary cells.^12^ Using an extralabyrinthine approach, the labyrinth is not open, and the electrodes are placed directly on the nerves.^13^

The evolution of knowledge about a theme in medicine requires Systematic Reviews (SR) to capture, recognize and synthesize the scientific evidence to support the proposals of qualified clinical practices in health.^19^ The purpose of this SR is to identify evidence in the scientific literature that VI in people with vestibular deficit improves vestibular function.

## Methods

In this study, a Systematic Review (SR) was performed, since it is a recognized methodology for high-level scientific research in the health area. The first step in starting an SR is to formulate a specific research question that contains the target population, the intervention or exposure, a comparison (if applicable), the expected outcomes and the types of study that may answer the question (PICOS).^19^ In this SR, the following question was posed: Do people with vestibular loss who use VI experience an improvement in vestibular function?

### Search strategy

The search strategy that was performed in this SR followed the criteria recommended by the Preferred Reporting Items for Systematic Reviews and Meta-Analyses—PRISMA^19^ and for which the protocol was registered on June 6, 2018 at the International Prospective Register of Systematic Reviews—PROSPERO^20^ under registration number CRD42018093469.

The search was performed in the PubMed, Scopus, Lilacs, Livivo and Speech bite databases. The gray literature was consulted through the Google Scholar database. There was no restriction on the period or language of publication.

The keywords of the search strategy were described as follows: “vestibular loss” OR “vestibular deficit” OR “vestibulopathy” OR “vestibular dysfunction” OR “vestibular deficiency” AND “vestibular implant” OR “vestibular prosthesis” OR “neural prosthesis” AND “vestibular function.” This search strategy was adapted to the other databases, where the correspondents were applied in Portuguese and Spanish in Lilacs. Then, a manual search of the references of the selected articles was performed.

After the search, the references of each database were exported to the EndNote X8 program (https://endnote.com/) and then these same references were exported from EndNote X8 to the Rayyan QCRI program (https://rayyan.qcri.org/). The purpose of these two programs was to record all duplicate articles that have been found in the scientific literature, promoting greater reliability in the selection of articles and proceeding to the eligibility stage.

### Eligibility criteria

The PICOS strategy (Population, Intervention, Comparison, Outcomes) was used to define the eligibility criteria.^19^ As inclusion criteria, we selected people with vestibular loss who used a VI, having as their intervention the vestibular prosthesis and as a comparison the period before and after surgery of the VI in each individual. The results had to contain an evaluation of vestibular function, and the types of studies should be prospective clinical studies, clinical cases and case reports. Exclusion criteria included patients who were not candidates for vestibular implantation, animal and in vitro studies and studies with abstention from postoperative data.

All studies were analyzed for eligibility in the screening phases based on the inclusion and exclusion criteria. In the first phase, all the studies were selected based on the title and abstract by two reviewers independently. There was no disagreement among the reviewers, ruling out the need to consult the third reviewer. In the case of summary abstention, but with an applicable title, the study was included in the second phase.

In the second phase, the same two reviewers read the full text of each selected article using the same inclusion and exclusion criteria, but adding the exclusion justification for each discarded study. The studies that met the inclusion criteria in this second step were included in a qualitative synthesis, and each type of study was analyzed according to the bias risk assessment of the Joanna Briggs Institute (JBI)^21^ through the JBI Critical Appraisal Checklist for Quasi-Experimental Studies (non-randomized experimental studies) and the JBI Critical Appraisal Checklist for Case Reports.

In the JBI Critical Appraisal Checklist, each question should be answered through four options: Yes (Y), No (N), Unclear (U) and Not Applicable (NA). The bias risk percentage calculation is done by the amount of “Y” that has been selected in the checklist. When “NA” was selected, this question was not considered in the calculation, according to the guidelines of the Joanna Briggs Institute.^21^ Up to 49% is considered a high risk of bias. From 50% to 70% is moderate and above 70% is low risk of bias.

After these evaluations, the selected studies underwent a statistical analysis to verify the possibility of constructing a meta-analysis. This analysis combines and summarizes results from multiple studies, thus increasing the accuracy and the power of evidence of the results.

## Results

**Fig. 1:**
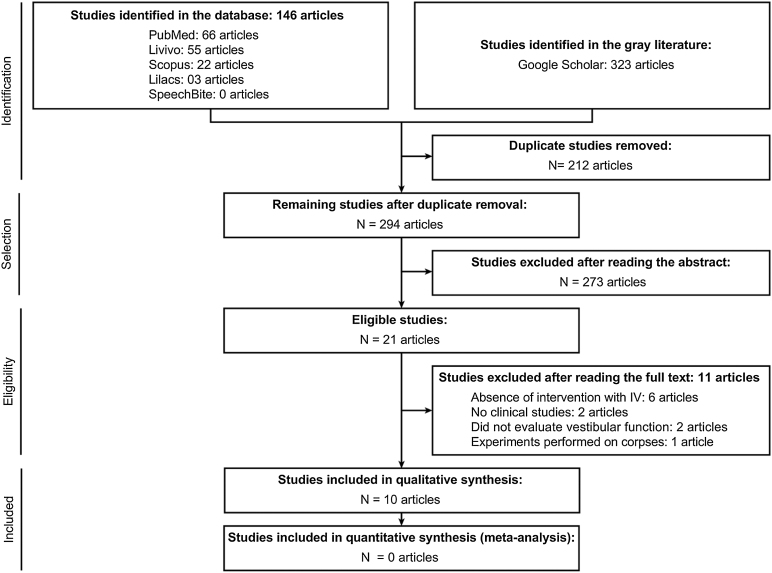
Diagram of the identification and selection of articles adapted from PRISMA.

In the first phase of this SR, 146 articles were found in five databases and 323 articles in the gray literature. After elimination of 212 duplicate studies, 294 were selected to read titles and abstracts. Of these, 273 were excluded by the established exclusion criteria. Of the 21 articles included in the second stage, which consisted of reading the full text of each study, 11 were excluded for the following reasons: in six articles,^12^, ^18^, ^22^, ^23^, ^24^, ^25^ they did not intervene with VI; two^2^, ^26^ were not clinical studies; two^27^, ^28^ did not assess vestibular function and in one^29^ study, the experiment was conducted using cadavers. In the end, 10 studies were selected for the qualitative analysis in the present SR (Table 1). No studies were found by performing a manual search of the references of the articles. The whole process of article selection is described in Fig. 1, which shows the flow PRISMA diagram for inclusion.

**Table 1:** Selected studies following the inclusion and exclusion criteria established in the systematic review.

	Title	Author	Location	Study design	N
1	Adaptation to steady-state electrical stimulation of the vestibular system in humans	Guyot et al.^30^	Geneva (Switzerland)	Case report	1
2	Postural responses to electrical stimulation of the vestibular end organs in human subjects	Phillips et al.^31^	Washington (EUA)	Quasi-experimental studies	4
3	Prosthetic implantation of the human vestibular system	Golub et al.^10^	Washington (EUA)	Case report	1
4	Artificial balance-restoration of the vestibulo-ocular reflex in humans with a prototype vestibular neuroprosthesis	Fornos et al.^8^	Geneva (Switzerland) e Maastricht (Netherlands)	Quasi-experimental studies	3
5	First functional rehabilitation via vestibular implants	Pelizzone et al.^3^	Geneva (Switzerland)	Quasi-experimental studies	3
6	Vestibular implants—Hope for improving the quality of life of patients with bilateral vestibular loss	Guinand et al.^32^	Geneva (Switzerland)	Quasi-experimental studies	11
7	Vestibular implants—8 years of experience with electrical stimulation of the vestibular nerve in 11 patients with bilateral vestibular loss	Guinand et al.^1^	Geneva (Switzerland) e Maastricht (Netherlands)	Quasi-experimental studies	11
8	The vestibular implant—frequency-dependency of the electrically evoked vestibulo-ocular reflex in humans	Van de Berg et al.^16^	Geneva (Switzerland)	Quasi-experimental studies	7
9	Vestibular implantation and longitudinal electrical stimulation of the semicircular canal afferents in human subjects	Phillips et al.^13^	Washington (EUA)	Quasi-experimental studies	4
10	The video head impulse test to assess the efficacy of vestibular implants in humans	Guinand et al.^33^	Geneva (Switzerland)	Quasi-experimental studies	3

Regarding the general characteristics of the included articles, the first published to perform VI in a human patient was in 2011;^30^ seven articles^1^, ^3^, ^8^, ^16^^,^^30^, ^32^, ^33^ were authored by the Geneva-Maastricht group and three^10^, ^13^, ^31^ were authored by the Washington group. Overall, these two groups performed the VI surgery in 18 people, ranging in age from 34 to 76 years (Table 2). The Geneva-Maastricht group performed its first vestibular implant in 2007 in a 68-year-old man. This group performed two types of VI surgery in 14 patients with vestibular loss of different etiologies, with six patients implanted through the extralabyrinthine approach and eight with an intralabyrinthine approach. The Washington group performed its first VI in a 56-year-old man. According to the publications, this group used the intralabyrinthine approach for implantation in four patients diagnosed with unilateral Meniere's disease.

**Table 2:** Demographics and implant details for each patient with bilateral vestibular hypofunction.

Patient	n of article	Sex	Age	BLV etiology	Deafness	Implanted ear	Year	Surgical approach	Tested vestibular electrodes (n of article)	Location
S1	6; 7; 8	M	68	Idiopathic	B	Left	2007	EL	PAN – 6; 7; 8	Geneva
S2	5; 6; 7; 8; 10	M	46	Idiopathic	B	Left	2008	EL	PAN – 5; 6; 7; 8; 10	Geneva
S3	6; 7	M	34	Idiopathic	B	Right	2008	EL	PAN – 6; 7	Geneva
S4	6; 7	M	71	Menière	B	Left	2011	EL	PAN – 6; 7	Geneva
S5	6; 7	M	63	Trauma	U	Right	2012	EL	PAN/LAN – 6; 7	Geneva
S6	6; 7; 8	F	48	Meningitis	U	Right	2012	IL	PAN/LAN/SAN – 6; 7	Geneva
									PAN ‒ 8	
S7	6; 7; 8	M	67	DFNA9	B	Left	2012	IL	PAN/LAN/SAN – 6; 7	Geneva
									SAN/LAN ‒ 8	
S8	4; 6; 5; 7; 8	M	66	DFNA9	B	Left	2013	IL	LAN ‒ 4	Maastricht
									PAN/LAN/SAN – 6; 5; 7; 8	
S9	4; 6; 7; 8; 10	F	67	Trauma	B	Left	2013	IL	LAN – 4; 10	Geneva
									SAN/LAN - 8	
									PAN/LAN/SAN – 6; 7	
S10	6; 7	M	64	DFNA9	B	Left	2013	IL	PAN/LAN/SAN – 6; 7	Geneva
S11	6; 7; 8	F	68	DFNA9	B	Left	2013	IL	PAN/LAN/SAN – 6; 7	Geneva
									SAN/PAN ‒ 8	
S12	4; 5	F	58	Meningitis	U	Right	‒	IL	LAN ‒ 4	Maastricht
									PAN/LAN/SAN ‒ 5	
S13	10	M	53	Trauma	‒	Right	2015	IL	SAN ‒ 10	Geneva
S14	1	M	69	Idiopathic	B	Left	‒	EL	PAN ‒ 4	Geneva
S15	2; 3; 9	M	56	Menière	U	Right	‒	IL	PAN/LAN/SAN – 2; 3; 9	Washington
S16	2; 9	M	76	Menière	U	Right	‒	IL	PAN/LAN – 2; 9	Washington
S17	2; 9	F	65	Menière	U	Right	‒	IL	PAN/LAN/SAN – 2; 9	Washington
S18	2; 9	F	72	Menière	U	Right	‒	IL	PAN/LAN/SAN – 2; 9	Washington

Age, Age at implantation; Year, Year of implantation; B, Bilateral; U, Unilateral; EL, Extralabyrinthine; IL, Intralabyrinthine; PAN, Posterior Ampullary Nerve; SAN, Superior Ampullary Nerve; LAN, Lateral Ampullary Nerve.

Since there is a non-negligible risk of inducing profound hearing loss with implantation surgery, in the cases of subjects with unilateral deafness, the VI was implanted in the same affected ear.

All ten articles were analyzed using the JBI Critical Appraisal Checklist for Quasi-Experimental Studies (Table 3) and the JBI Critical Appraisal Checklist for Case Reports (Table 4) according to each type of study, and nine articles^3^, ^8^, ^10^, ^13^^,^^16^, ^30^, ^31^, ^32^, ^33^ showed low risk of bias and one article^1^ showed moderated risk of bias.^21^

**Table 3:** JBI Critical appraisal checklist for quasi-experimental studies.

	Phillips et al.^31^	Fornos et al.^8^	Pelizzone et al.^3^	Guinand et al.^32^	Guinand et al.^1^	Van de Berg et al.^16^	Phillips et al.^13^	Guinand et al.^33^
Is it clear in the study what is the “cause” and what is the “effect” (i.e. there is no confusion about which variable comes first)?	Y	Y	Y	Y	Y	Y	Y	Y
Were the participants included in any comparisons similar?	Y	Y	Y	Y	Y	Y	Y	Y
Were the participants included in any comparisons receiving similar treatment/care, other than the exposure or intervention of interest?	Y	Y	NA	N	N	Y	Y	N
Was there a control group?	Y	Y	Y	Y	N	Y	Y	Y
Were there multiple measurements of the outcome both pre and post the intervention/exposure?	Y	Y	Y	N	N	N	Y	Y
Was follow up complete and if not, were differences between groups in terms of their follow up adequately described and analyzed?	Y	Y	Y	Y	Y	Y	Y	Y
Were the outcomes of participants included in any comparisons measured in the same way?	Y	Y	Y	Y	NA	Y	Y	Y
Were outcomes measured in a reliable way?	Y	Y	U	Y	Y	Y	Y	Y
Was appropriate statistical analysis used?	Y	Y	Y	Y	Y	Y	Y	Y
Bias risk (%)	100%	100%	87.5%	77.77%	62.5%	88.88%	100%	88.8%

Y, Yes; N, No; U, Unclear; NA, Not applicable.

**Table 4:** JBI Critical appraisal checklist for case reports.

	Guyot et al.^30^	Golub et al.^10^
Were patient’s demographic characteristics clearly described?	Y	Y
Was the patient’s history clearly described and presented as a timeline?	N	Y
Was the current clinical condition of the patient on presentation clearly described?	Y	Y
Were diagnostic tests or assessment methods and the results clearly described?	Y	Y
Was the intervention(s) or treatment procedure(s) clearly described?	Y	Y
Was the post-intervention clinical condition clearly described?	Y	Y
Were adverse events (harms) or unanticipated events identified and described?	Y	Y
Does the case report provide takeaway lessons?	Y	Y
Bias risk (%)	87.5%	100%

Y, Yes; N, No.

It was not possible to elaborate a meta-analysis in this SR because the articles included presented very different methodologies and measures resulting from the tests.

Guyot et al.^30^ conducted a case study assessing a patient with idiopathic bilateral deafness and vestibular loss who was already scheduled for cochlear implantation and received a VI. They assessed whether the patient could adapt to continuous electrical stimulation of the vestibular system and whether it was possible to elicit artificial eye movements via modulation of the stimulation. Successive “on-off” cycles of continuous electrical stimulation in the vestibular electrodes resulted in nystagmic response with a progressively shorter duration. Once the adapted state was reached upon constant stimulation, amplitude or frequency modulations of electrical stimulation produced smooth oscillatory conjugated eye movements.

Phillips et al.^31^ performed VI in four patients with intractable unilateral Meniere’s disease in the right ear. All subjects were implanted with the VI (UW/Nucleus) based on the Nucleus Freedom cochlear implant (Cochlear, Ltd.). During surgery, an electrode array was inserted into the perilymphatic space adjacent to the ampulla of each of the three semicircular canals via a small fenestration in the bony labyrinth adjacent to the ampullae. The fenestrations were closed with fascia while trying not to occlude the canal lumens, and the leads external to the fenestrations were secured with suture. During array placement, electrically Evoked Compound Action Potential (vECAP) recordings were obtained to optimize electrode placement for afferent activation. If large amplitude vECAPs were not obtained at low current levels, the fenestration was widened and the electrode array moved closer to the ampulla. A remote ground ball electrode was placed under the temporalis muscle. In three subjects, the surgery was performed with stimulating electrode arrays in three semicircular canals of the affected right ear, and one subject was implanted with arrays in the lateral and posterior canals only. Prior to the electrical stimulation studies, before and after device implantation, the patients received a battery of clinical vestibular tests to evaluate their underlying vestibular function, including calorics, rotary chair, dynamic visual acuity, subjective visual vertical testing and posturography. Testing was conducted over two sessions, 17–136 weeks after surgical implantation. Data analysis performed with the system offline showed that all subjects had significant decrease in caloric responses in the implanted ear postoperatively and showed a reduction from preoperative levels in rotary chair gain, an advance in phase and an asymmetry toward the implanted ear postoperatively.

Golub et al.^10^ described the case of a patient with uncontrolled Ménière’s disease submitted to VI. At 6 weeks post-implantation, the prosthesis was programmed with a take-home map. Nine level settings were provided in increments of 25 μA, ranging from 150 μA to 350 μA. The patient was instructed to progress through the map settings during an acute attack until the symptoms were minimized or eliminated. At about the time his take-home processor was provided, his attacks ceased. He had only one subsequent attack, which occurred 6 months postoperatively. Turning off the implant also resulted in increased symptoms. Comparative to preoperatively values, rotational chair velocity step testing revealed decreased gain at 6 weeks post-implantation. At 63 weeks post-implantation, the gain recovered, but there was a persistent asymmetry. Caloric testing indicated a 26% unilateral weakness preoperatively, which increased to 95% at 6 weeks postoperatively and recovered slightly to 71% at 87 weeks. Electrical stimulation resulted in canal-specific eye movements, although the thresholds increased over time.

Fornos et al.^8^ investigated whether VI was possible to artiﬁcially restore the VOR in three patients with Bilateral Vestibular Loss (BVL). The etiology of these patients was meningitis (F-58), DFNA9 (M-66) and traumatic (F-67) and they were recruited at the Service of Otorhinolaryngology and Head and Neck Surgery at the Geneva University Hospitals and at the Division of Balance Disorders at the Maastricht University Medical Center. These subjects received a vestibular neuroprosthesis prototype consisting of a modiﬁed cochlear implant (MED-EL, Innsbruck, Austria) and the implantation was performed using an intralabyrinthine surgical approach. This device, in addition to the cochlear electrode array, provided extracochlear electrodes, which were implanted in the vicinity of the ampullary branches of the vestibular nerve. Patients were submitted to horizontal whole-body rotations (i.e., around the vertical axis) in complete darkness (without head stabilization). Rotation velocities had a sinusoidal proﬁle with a 30°/s peak amplitude based on the typical motion proﬁle of human locomotion. Five different rotation frequencies (0.1, 0.25, 0.5, 1 and 2 Hz) were tested. Rotations were achieved with a custom-modiﬁed, velocity controlled rotatory chair. Tests were performed without any electrical stimulation (system OFF) and upon electrical stimulation of the lateral semicircular canal (system ON). In the system ON condition, the amplitude of the electrical stimulation was modulated via the motion signal captured by an inertial sensor (gyroscope). Signiﬁcantly higher VOR responses were observed when the prototype was turned ON. Furthermore, VOR responses increased signiﬁcantly as the intensity of the stimulation increased, reaching on average 79% of those measured in healthy volunteers in the same experimental conditions.

Pelizzone et al.^3^ performed VI in three patients with profound bilateral deafness and vestibular loss. First, the patient received steady state electrical stimulation to restore an artificial “spontaneous” firing rate in his deafferented vestibular nerve until the nystagmic responses vanished. Once the patient was in this “adapted state”, a gyroscope, to code rotational movements, was used to up- and down-modulate the amplitude of the train of pulses delivered to the vestibular electrode. The patient’s eye movements were recorded while the patient was submitted to whole body rotations in the horizontal plane in complete darkness. The results showed that in peak angular velocity at 1 Hz, the VOR gain of the 3 tested patients was very low in the system offline (gains <0.2). In the system on condition, the gains in the 3 patients improved significantly and reached up to 75–98% of the median VOR gain of healthy volunteers. Furthermore, the gain increased significantly as we increased the intensity of stimulation. The VOR gain in the system offline was very low, while it increased significantly in the system on condition, reaching 51–69% of that observed in healthy volunteers.

Guinand et al.^32^ described eleven BVL patients with bilateral or unilateral severe deafness, all of whom were implanted with a VI developed through modified a CI providing one to three extracochlear electrodes. During surgery, the cochlear array was inserted, and each extracochlear branch was placed in proximity to the Posterior (PAN), Lateral (LAN), and Superior (SAN) ampullary branches of the vestibular nerve. In total, 24 vestibular electrodes were implanted in these patients. An extralabyrinthine surgical approach was performed in five patients and an intralabyrinthine approach was used in six patients. Device activation took place no earlier than four weeks postoperatively, when healing of the surgery site was assumed to be complete. Videonystagmography (VNG) and Electronystagmography (ENG) were used for vestibular tests. HIT was performed with the Ulmer system (Synapsis©; Marseille, France), the EyeSeeCam (EyeSeeCam VOG©; Munich, Germany) and/or the ICS Impulse (Otometrics, Denmark). Cochlear electrodes were always switched off during the experimental procedure. The two-dimensional eye-in-head angular position was recorded using a fast monocular 2D video oculography system (EyeSeeCam VOG; Munich, Germany). All eye movement recordings were performed in the dark with the patients sitting in an upright position. No complications related to surgery or to the experimental procedure were reported. Despite different etiologies (Idiopathic, Menière’s, Traumatic, Meningitis and DFNA9) and different disease durations, it was possible to elicit controlled eye movements of variable amplitudes and directions in all eleven BVL patients up to almost 8 years after implantation. These results indicate that electrical stimulation of the vestibular nerve has a significant functional impact; eye movements generated this way could be sufficient to restore gaze stabilization during essential everyday tasks such as walking. However, the results also demonstrate that electrically evoked eye movements result in a significant loss of visual acuity. Importantly, the goal of the vestibular implant is not to impair visual acuity, but the opposite. From the time a fine-tuned and an appropriate match between pacing and patient movement were made, the possibility of improvement in visual acuity is real and this would improve the stabilization of the images in the retina.

In a posterior study of the same 11 patients, Guinand et al.^1^ did not assess visual acuity as was done in Guinand et al.^32^ Guinand et al.^1^ had a total of 24 vestibular electrodes available for electrical stimulation in these 11 patients. Of these, three electrodes were unresponsive, so a vestibular threshold was determined in only 21 of the 24 available electrodes. In 19 of these electrodes, the upper comfortable level corresponded to facial nerve stimulation. In 2 electrodes, no upper comfortable level was determined even at the highest current amplitude tested (550 mA). The two-dimensional eye-in-head position was recorded using a fast monocular 2D video oculography system (EyeSeeCam VOG) to assess the effects of the electrical stimulation. All eye movement recordings were done in darkness with patients sitting in an upright position with electrical stimulation in the labyrinth to capture the eVOR. Consistent with the very variable dynamic ranges measured, the range of eye velocities was also very variable. The mean peak velocities per electrode across patients were 8.7–7.6°/s for PAN (n = 11), 13–12.5°/s for LAN (n = 6), and 11.9–6.6°/s for SAN (n = 5). The patients diagnosed with DFNA9 showed the smallest responses. One of the results of this study is that eye movements could be successfully evoked in a heterogeneous group of patients regarding the etiology of the deficit or the duration of the disease.

Van de Berg et al.^16^ investigated how the characteristics of the eVOR changed as a function of the modulation frequency in patients with a VI, and compared these results to the “natural” VOR responses obtained in healthy age-matched volunteers who were subjected to horizontal whole body rotations with equivalent sinusoidal velocity proﬁles at the same frequencies. Seven patients with BVL received a VI prototype consisting of a modiﬁed cochlear implant (MED-EL, Innsbruck, Austria) with extracochlear branches for vestibular stimulation. Twelve vestibular electrodes implanted at different anatomical locations were tested: four electrodes implanted in the vicinity of the superior ampullary nerve, three electrodes implanted in the vicinity of the lateral ampullary nerve and ﬁve electrodes implanted in the vicinity of the posterior ampullary nerve. The intensity of modulation was kept constant throughout the experimental trials and 3 modulation frequencies (0.5, 1 and 2 Hz) were tested. The eVOR experiments were conducted under stationary conditions and the electrical stimulation was delivered exclusively to one vestibular electrode at a time. Individual and pooled results revealed a clear frequency-dependent behavior for the three stimulation sites. They observed a strong and signiﬁcant effect of frequency in the total peak eye velocity of the eVOR: the total peak eye velocity increased with increasing frequency for both groups, without any signiﬁcant effect between the groups. Other characteristics of the eVOR (angle, habituation-index and asymmetry) showed no signiﬁcant frequency-dependent effect. This effect was similar to that observed in the “natural” VOR. The eVOR angle was markedly variable across the BVL Group for the whole tested frequency range. These results demonstrated that, at least in the speciﬁc (limited) frequency range tested, the VI closely mimics the natural frequency-dependency of the vestibular system.

Phillips et al.^13^ implanted four subjects with intractable unilateral Meniere’s disease. Although the subjects had limited vestibular function and relatively poor hearing pre-surgically, following implantation of the VI, the subjects lost hearing and vestibular function in the implanted ear relative to their preoperative levels with no recovery of vestibular function. Additionally, the electrical stimulation of VI appeared to be well tolerated by the patients, with no signiﬁcant pain, nausea or electrically evoked auditory percepts in association with the electrical stimulation. Overall, the vECAP recording results showed an increase in the horizontal slow phase velocity with an increasing stimulus rate. The authors also observed a general decrease over time in the slow phase velocity elicited by electrical stimulation in most canals in these four subjects with Meniere’s disease.

Guinand et al.^33^ evaluated three patients suffering from severe BVL and implanted with a VI. The high-frequency VOR was assessed using the vHIT, while motion-modulated electrical stimulation was delivered via one of the implanted vestibular electrodes at a time. In three out of the five tested electrodes, the VOR gain increased monotonically with increased stimulation strength when head impulses were delivered in the plane of the implanted canal. In the three cases presented, the VOR responses measured without activation of the VI were deficient in both directions of the tested plane.

## Discussion

The present SR was designed to identify evidence in the scientific literature that VI in people with BVL improves vestibular function.

We observed that the intralabyrinthine approach was most frequently used, probably because the extralabyrinthine approach is surgically more challenging with regard to the extent of the exact site of stimulation. However, the main disadvantage of the intralabyrinthine approach is the risk of hearing compromise when the labyrinth is opened and the electrodes are inserted into the semicircular canals.^1^ For this reason, the Geneva-Maastricht group chose to perform surgery only in deaf patients in the implanted ear. The Washington group underwent surgical implantation^10^, ^13^, ^31^ in four Menière’s patients with residual hearing: all of them lost their hearing. However, these negative results do not necessarily imply that hearing will always be affected by the implantation of electrodes in the semicircular canals. Research on rhesus monkeys has shown that hearing preservation is possible with the intralabyrinthine approach.^9^, ^14^, ^34^, ^35^ Therefore, the main challenge of future studies is to optimize the surgical technique and to develop electrodes that can elicit effective stimulation for the activation of vestibular function and, simultaneously, the preservation of hearing in patients.

The Geneva/Maastricht team showed that, by using a virtual motion profile to modulate the baseline electrical stimulation of the ampullary branches of the vestibular nerve, it was possible to artificially generate eye movements in all BVL patients of their group, independently of the disease etiology, duration of implantation, and model of the vestibular implant prototype used. Their results also demonstrate that electrically evoked eye movements resulted in a significant loss of visual acuity.

The group at the University of Washington showed that implantation of the device in four Meniere’s patients produced hearing and vestibular loss in the implanted ear. These results suggest that electrical stimulation of the vestibular end organ in human subjects provided controlled vestibular inputs over time, but in Meniere’s patients this result was apparently obtained at the cost of hearing and vestibular function in the implanted ear.

We also observed better artificial VOR responses at higher rotation frequencies. At rotation frequencies of 0.1 and 0.25 Hz, the artificially evoked VOR was practically absent. The VOR response started to grow at 0.5 Hz, reaching a maximum performance at rotation frequencies of 1 and 2 Hz. This phenomenon resembles well-known and documented dynamic characteristics of the normal VOR. Important everyday activities such as walking induce head movements predominantly in the 1–2 Hz frequency range. This artificial restoration of the VOR in these implanted patients can be therefore considered as substantial functional recovery. Furthermore, this achievement was possible for patients with substantially different deficit durations and with different BVL etiologies.

This SR showed that humans can adapt to electrical stimulation of the vestibular system without too much discomfort and that 77% of the 18 implanted patients experienced an improvement in vestibular function.

## Conclusion

Several studies have demonstrated these findings support the feasibility of a VI to restore the VOR in a broad frequency range and illustrated new challenges for the development of this technology to guarantee an improvement in the vestibular function without losing hearing.

Taken together, this evidence holds good promise for achieving the first real alternative for rehabilitating patients with a BVL and warrants further research efforts and increased interest in the field.

## Conflicts of interest

The authors declare no conflicts of interest.
